# Crystal Structures of *T. b. rhodesiense* Adenosine Kinase Complexed with Inhibitor and Activator: Implications for Catalysis and Hyperactivation

**DOI:** 10.1371/journal.pntd.0001164

**Published:** 2011-05-24

**Authors:** Sabine Kuettel, Jason Greenwald, Dirk Kostrewa, Shaheen Ahmed, Leonardo Scapozza, Remo Perozzo

**Affiliations:** 1 Pharmaceutical Biochemistry Group, School of Pharmaceutical Sciences, University of Geneva, University of Lausanne, Lausanne, Switzerland; 2 Laboratory of Physical Chemistry, ETH Zurich, Zurich, Switzerland; 3 Gene Center Munich, Department of Biochemistry, Ludwig-Maximilians-University Munich, Munich, Germany; Institut Pasteur de Montevideo, Uruguay

## Abstract

**Background:**

The essential purine salvage pathway of *Trypanosoma brucei* bears interesting catalytic enzymes for chemotherapeutic intervention of Human African Trypanosomiasis. Unlike mammalian cells, trypanosomes lack de novo purine synthesis and completely rely on salvage from their hosts. One of the key enzymes is adenosine kinase which catalyzes the phosphorylation of ingested adenosine to form adenosine monophosphate (AMP) utilizing adenosine triphosphate (ATP) as the preferred phosphoryl donor.

**Methods and Findings:**

Here, we present the first structures of *Trypanosoma brucei rhodesiense* adenosine kinase (TbrAK): the structure of TbrAK in complex with the bisubstrate inhibitor P^1^,P^5^-di(adenosine-5′)-pentaphosphate (AP5A) at 1.55 Å, and TbrAK complexed with the recently discovered activator 4-[5-(4-phenoxyphenyl)-2*H*-pyrazol-3-yl]morpholine (compound **1**) at 2.8 Å resolution.

**Conclusions:**

The structural details and their comparison give new insights into substrate and activator binding to TbrAK at the molecular level. Further structure-activity relationship analyses of a series of derivatives of compound **1** support the observed binding mode of the activator and provide a possible mechanism of action with respect to their activating effect towards TbrAK.

## Author Summary

Recently, we discovered that 4-[5-(4-phenoxyphenyl)-2*H*-pyrazol-3-yl]morpholine (compound **1**) and its derivatives exhibit specific antitrypanosomal activity toward *T. b. rhodesiense*, the causative agent of the acute form of HAT. We found that compound **1** would target the parasite adenosine kinase (TbrAK), an important enzyme of the purine salvage pathway, by acting via hyperactivation of the enzyme. This represents a novel and hitherto unexplored strategy for the development of trypanocides. These findings prompted us to investigate the mechanism of action at the molecular level. The present study reports the first three-dimensional crystal structures of TbrAK in complex with the bisubstrate inhibitor AP5A, and in complex with the activator (compound **1**). The subsequent structural analysis sheds light on substrate and activator binding, and gives insight into the possible mechanism leading to hyperactivation. Further structure-activity relationships in terms of TbrAK activation properties support the observed binding mode of compound **1** in the crystal structure and may open the field for subsequent optimization of this compound series.

## Introduction

Human African Trypanosomiasis (HAT), also known as sleeping sickness, belongs to the most neglected diseases. It is distributed throughout sub-Saharan Africa [1], [2] and affects about 0.5 million people on the African continent, causing 50′000 to 70′000 deaths per year [3]. The therapy for this fatal disease relies on a few drugs which are associated with severe side effects. Moreover, an increase of resistance to these drugs has been observed in several foci particularly in central Africa. Thus, novel targets and new efficacious chemotherapeutic agents are urgently needed [4].

To develop a rational approach for chemotherapeutic intervention of HAT, it is crucial to find differences within the biochemical pathways of the parasite with respect to the host. Therefore, parasite purine salvage offers attractive opportunities due to the fact that, unlike mammalian cells, trypanosomes lack de novo purine synthesis and completely rely on salvage from their hosts [5], [6]. The trypanosomal salvage pathway is very versatile and is capable of incorporating any physiological purine base and nucleoside, and of interconverting the corresponding nucleotides [7]. However, nucleoside uptake in *T. brucei* is most efficient for adenosine and occurs via high affinity transporters that accumulate adenosine in the cell [8], [9]. Moreover, adenosine is incorporated faster than any other nucleoside [7]. Recently *T. brucei* adenosine kinase (TbAK) was identified as a key enzyme mediating high affinity adenosine salvage in the purine salvage pathway [10].

Adenosine kinase (EC 2.7.1.20, ATP:adenosine 5′-phosphotransferase) catalyzes the phosphorylation of adenosine to adenosine monophosphate (AMP) in presence of Mg^2+^ and utilizing ATP as the preferred phosphoryl donor. A common property of adenosine kinases from various organisms is their control via substrate-inhibition to prevent non-physiologically high intracellular purine nucleotide levels [11], [12], [13], [14], and recently it has been shown that TbrAK activity is strongly inhibited by its substrate adenosine [10], [15]. Adenosine kinases belong to the phosphofructokinase B family of carbohydrate kinases, which includes ribokinase, inosine-guanosine kinase, fructokinase, and 1-phosphofructokinase. The members of this family are characterized by the presence of two sequence motifs which are a highly conserved di-glycine switch positioned near the N-terminus and a DXNGAGD peptide sequence located near the C-terminus [16].

To date, adenosine kinases from several sources have been described and the structures of three organisms have been reported: (*Homo sapiens* [17], [18], *Toxoplasma gondii* [19], [20], [21], [22], *Mycobacterium tuberculosis* [23], [24]). The available structures include the apo forms as well as complexes with substrates and inhibitors. The corresponding adenosine kinase of *T. b. rhodesiense* (TbrAK) is a 345-residue (37.9kDa) enzyme that functions as a monomer, a common characteristic of other adenosine kinases analyzed to date, including the enzyme in *T. gondii* (TgoAK) and the human homologue (HsaAK). Interestingly, the adenosine kinase of *M. tuberculos*is (MtuAK) is reported to form a functional dimer [13].

In general, the sequence identity among adenosine kinases of different species is moderate (17% to 40%), however their structures are remarkably similar. The enzyme consists of two domains of which the small lid domain is formed by a five-stranded α/β motif, whereas the large domain is composed of a mixed α/β structure reminiscent of the Rossmann fold [25]. The active site of the enzyme is located at the domain interfaces, with adenosine binding in a deeply buried cavity and covered by the small lid domain. The ATP binding site is located at an adjacent site in the large domain with the γ-phosphate group pointing near the 5′-end of the ribose moiety of adenosine.

Recent crystallographic studies of TgoAK in presence and absence of substrate [20] have revealed that adenosine kinases appear in two different conformations. The apo form of the enzyme is found to be in the open conformation, which upon binding of adenosine, closes by a rotation of 30° of the lid domain relative to the large domain. This will sequester adenosine and at the same time initiate the formation of the ATP binding site in the large domain. Binding of ATP further induces local structural changes and leads to the formation of the anion hole. Once ATP has bound, the completely closed conformation is achieved and the catalytically important residues are positioned in the correct orientation for catalytic transformation. This catalytic mechanism is consistent with most recent studies postulating an ordered bi-bi kinetic mechanism [14], [26], [27], [28].

Recently, we described 4-[5-(4-phenoxyphenyl)-2*H*-pyrazol-3-yl]morpholine (compound **1**) as a potent trypanocidal exhibiting an IC_50_ of 1 µM towards *Trypanosoma brucei rhodesiense* (*T. b. rhodesiense*) while exhibiting low cytotoxicity [15], [29]. Using a chemical proteomics approach we identified adenosine kinase of *T. b. rhodesiense* (TbrAK) as its putative target [15]. This finding was confirmed by RNA interference experiments and drug sensitivity tests, and further biochemical validation demonstrated that compound **1** interacts specifically and tightly with TbrAK. Kinetic analysis revealed that compound **1** enhanced enzyme activity by abolishment of the intrinsic substrate-inhibition, suggesting that uncontrolled activity (hyperactivity) of TbrAK may be the putative mechanism of action leading to cell toxicity [15].

Understanding the mechanism of action on a molecular level of substrate and activator binding is important for the development of this novel trypanocidal strategy and for future improvement of compound **1** in terms of pharmacokinetic and pharmacodynamic aspects. To this end, we have solved the structure of TbrAK in complex with the bisubstrate inhibitor P^1^,P^5^-di(adenosine-5′)-pentaphosphate (AP5A) at 1.55 Å resolution, and TbrAK complexed with the activator 4-[5-(4-phenoxyphenyl)-2*H*-pyrazol-3-yl]morpholine (compound **1**) at 2.8 Å resolution. In addition, we have analyzed the activating effects of a series of derivatives of compound **1**. We discuss the results in terms of structure-activity-relationship and conclude that it supports the observed binding mode of compound **1**.

## Methods

### Materials and instrumentation

HPLC separations were performed using a RP-18e (5 µm) cartridge (LiChroCART 250-4) protected with a guard column on a Merck instrument. CD and ITC measurements were accomplished on Jasco J-815 CD spectrophotometer and the VP-ITC microcalorimeter (Microcal Inc., Northampton, MA), respectively. Proteins were concentrated using Amicon ultra centrifugal filter devices from Milipore. Diadenosine pentaphosphate (AP5A) and all other chemicals were obtained from Sigma unless otherwise noted. Crystal screen solutions were from Hampton Research.

### Crystallization

The protein was cloned, expressed and purified exactly as described elsewhere [15]. The protein was concentrated to 10–15 mg/ml, mixed with an equal volume (2 µl) of precipitant solution and crystallized using the sitting drop vapor diffusion technique. Crystals of TbrAK in complex with AP5A were grown at 16°C in presence of 1 mM AP5A in 0.2 M sodium acetate trihydrate, 0.1 M sodium cacodylate, pH 6.0, and 24% w/v polyethylene glycol 8000 (crystal form 1), or in 0.1 M tri-sodium citrate dihydrate, pH 5.6, 20% v/v iso-Propanol, and 20% w/v polytethylene glycol 4000 (crystal form 2). Crystals grew to the maximum dimension of 150×50×50 µm within two to three weeks.

Crystals of apo TbrAK were grown at 4°C in 0.1 M Tris, pH 9.0 and 60% MPD and reached their maximum size of 50×20×20 µm after one to two weeks. Crystals of TbrAK complexed to compound **1** were prepared by directly adding compound **1** dissolved in mother liquor to drops containing apo TbrAK crystals.

### Data collection, structure solution and refinement

All data were collected from crystals flash frozen in a nitrogen stream at 100 K as consecutive series of 0.5° rotation images on beam line X06SA at the Swiss Light Source, SLS, in Villigen, Switzerland. The AP5A-TbrAK data were collected on a MAR225 CCD whereas data related to TbrAK crystals soaked with compound **1** were collected using the Pilatus 6 M detector. The AP5A data sets were processed with XDS [30] and the compound **1** data with MOSFLM [31]. Crystals of apo TbrAK did not afford interpretable diffraction patterns under any circumstances. In contrast, crystals of the TbrAK-compound **1** complex displayed non-merohedral twinning that rendered data processing possible by indexing one lattice via manually picking reflections in MOSFLM. After integration, the data were scaled with SCALA [32], leading to reasonable statistics considering the low I/sigma(I) (3.6 overall) of the data and the potential for overlap with other lattices (Table 1).

**Table 1:** Crystallographic data and refinement statistics.

	TbrAK-AP5A		TbrAK-compound 1
	Crystal form 1	Crystal form 2	
Space group	P2_1_ (No. 4)	P2_1_ (No. 4)	P4_1_2_1_2 (No. 92)
Unit cell dimensions			
a, b, c (Å)	88.4, 86.4, 89.4	68.9, 70.5, 72.4	61.2, 61.2, 193.8
α, β, γ (°)	90, 90.1, 90	90, 90.8, 90	90, 90, 90
Radiation wavelength	0.95 Å	1.00 Å	1.00 Å
Resolution range (Å)	80-2.3 (2.4-2.3)^e^	80-1.55 (1.60-1.55)^e^	58-2.8 (2.95-2.8)^e^
No. of unique reflections	56629	97047	9747
Redundancy overall	1.8 (1.7)^e^	3.8 (3.8)^e^	3.2 (3.3)^e^
R_sym_ overall^a^	0.065 (0.45)^e^	0.054 (0.44)^e^	0.17 (0.41)^e^
R_meas_ overall^b^	0.090 (0.62)^e^	0.063 (0.51)^e^	0.20 (0.48)^e^
Completeness overall	0.95 (0.98)^e^	0.97 (0.95)^e^	0.98 (0.98)^e^
No. of protein monomers in AU	4	2	1
No. of refined atoms			
Protein	―f	5319	2600
AP5A / compound **1**	―f	114	24
Na^+^	―f	2	0
Water	―f	626	24
R-factor / Free R-factor^c^	―f	0.18/0.21	0.23/0.29
RMSD bond lengths / angles^d^	―f	0.012 Å /1.5°	0.006 Å /1.0°
Ramachandran plot			
Preferred / allowed / outlier	―f	96.1% /3.6% /0.3%	94.9% /4.5% /0.6%

a R_sym_  =  ∑_h_∑_i_ |I_i_(h) − <I(h)>| / ∑_h_∑_i_ I_i_(h) , where I_i_(h) and <I(h)> are the i^th^ and mean intensity over all symmetry-equivalent reflections h.

b R_meas_  =  ∑_h_ (n_h_/n_h_−1)^½^ ∑_i_ |I_i_(h) − <I(h)>| / ∑_h_∑_i_ I_i_(h) , where I_i_(h) and <I(h)> are the i^th^ and mean intensity, and n_h_ is the multiplicity over all symmetry-equivalent reflections h [42].

c R =  ∑||F_O_ |− |F_C_|| / ∑|F_O_|, where |F_C_|is the calculated structure factor amplitude of the model, and |F_O_|is the observed structure factor amplitude; the Free R-factor was calculated against a random 5% test set of reflections that was not used during refinement.

d RMSD, root-mean-square deviation from the parameter set for ideal stereochemistry [43].

e Values in parentheses refer to the highest resolution shell.

f The model was only partially refined against this data set, but provided the search model for the refinement of the data set of crystal form 2 (see text for details).

The crystal structure of TbrAK bound with AP5A was initially solved by molecular replacement using the data set of crystal form 1 (Table 1). The results from the self-rotation function and from the native Patterson function were consistent with the hypothesis of a tetramer in the asymmetric unit with its fourfold non-crystallographic axis oriented parallel to the monoclinic b-axis. A BLASTP search of the TbrAK amino acid sequence against the Protein Data Bank [33] gave a top hit with p = 6·10^−69^ and a sequence identity of 40% for the human adenosine kinase, PDB entry code 1BX4 [17]. A search model for molecular replacement was constructed from PDB entry 1BX4 using CHAINSAW [32] with the option to keep different amino acids in the BLASTP sequence alignment up to the last common atom. Molecular replacement with PHASER [34] found four molecules in the asymmetric unit. The four molecules in the asymmetric unit form a tetramer with its fourfold axis parallel to the b-axis consistent with the self-rotation function and an x,z-displacement consistent with the native Patterson peak, confirming the initial tetramer hypothesis.

Iterative rounds of model building with COOT [35] and restrained maximum-likelihood refinement with REFMAC [36] resulted in a partially refined model for which most amino acids and the bound AP5A could be built into the electron density maps. One protein monomer with bound AP5A was used as search model for molecular replacement using the higher resolution data set of crystal form 2. A molecular replacement search with MOLREP [37] gave a clear solution with two molecules in the asymmetric unit. Iterative rounds of model building with COOT and TLS-refinement and restrained maximum-likelihood refinement with REFMAC resulted in the final model at 1.55 Å resolution described in Table 1.

To solve the structure of the TbrAK-compound **1** complex chain A from the TbrAK-AP5A structure was used as a model for the molecular replacement search in MOLREP. One molecule was located above the background signal. However, the statistics were not as good as would be expected for a search with the identical structure. Inspection of the electron density maps revealed that a region of the protein had moved relative to the majority of the structure. Manual repositioning of this small domain into the electron density was unambiguous and removed the observed clashes in the crystal contacts. The model was refined as before and the density for compound **1** was clearly visible in the adenosine binding site as the highest signal in the mFo-DFc map (5.5σ). Despite the moderate resolution of 2.8 Å, the orientation of compound **1** was unambiguously deduced by considering a) the better fit into the density without adopting sterically unfavorable conformations of the phenoxyphenyl moiety, and b) that only with the morpholine moiety pointing toward the protein could important hydrogen bonds between protein and compound be formed.

### Activity assay

The activation potential of a series of compound **1** derivatives (compounds **2**–**7**) was measured using a recently developed HPLC method [15]. The resulting ADP/ATP ratios were calculated and used as a measure of activity. For comparative reasons the activity recorded in absence of compound was set to 100%. The mean of three independent measurements is reported.

### Thermal stability assay

Thermal unfolding of TbrAK was studied by CD spectroscopy as outlined elsewhere [15]. Briefly, all measurements were done in a 0.5 mm cell and contained 0.1% DMSO for solubility reasons as well as 2.5 mM EDTA to prevent enzyme activity. TbrAK in a buffer containing 20 mM Hepes and 150 mM NaCl (pH 7.0) was kept constant at a final concentration of 0.5 mg/ml (13 µM). Thermal stability was analyzed in presence AP5A, adenosine, ATP (all at 500 µM), and compounds **1**–**7** (50 µM). For evaluation of the CD spectra, the buffer spectrum was subtracted from the protein CD spectrum. All thermal unfolding curves were fitted to a two-state model that had been published recently [38]. The mean of three independent experiments is reported.

### Isothermal titration calorimetry

Binding affinity constants for adenosine and ATP were determined using isothermal titration calorimetry (ITC). To this end, TbrAK was dialyzed into a buffer consisting of 20 mM Tris, 150 mM NaCl, and 5% glycerol (pH 7.5) and used at concentrations around 90 µM. The ligands were dissolved directly into the dialysis buffer to give a final concentration of around 2 mM. All solutions were filtered and thoroughly degassed before use. A typical titration experiment consisted of a first control injection of 1 µl followed by 50 to 60 injections, each of 5 µl (20 s duration), using a 4 min interval. Raw data were collected, corrected for ligand heats of dilution, integrated and fitted to the appropriate binding model using the Microcal Origin software supplied with the instrument. The measurements were performed in duplicate.

## Results and Discussion

### Crystallization and structure solution

Purified TbrAK [15] co-crystallized with the bisubstrate inhibitor AP5A in space group P2_1_ in two crystal forms which diffracted to 2.3 Å and 1.55 Å resolution (see Table 1 for crystallization statistics). The crystal structure of TbrAK was first solved by molecular replacement using the initial data set (2.3 Å) of crystal form 1 and human adenosine kinase (HsaAK; PDB entry code 1BX4) [17] as a search model. After the partially refined model was created, i.e. most amino acids and the inhibitor AP5A were built into the electron density map, the crystal form 2 became available and afforded a high resolution data set (1.55 Å). Subsequently, one subunit of the partially refined model was considered the most appropriate search model to solve the structure of the new high resolution data set. The two molecules (A and B) found in the asymmetric unit were not related by proper non-crystallographic symmetry and exhibited a mixed hydrophilic/hydrophobic contact interface typical for crystal contacts. Apparently, the molecules do not form a functional dimer and thus could be considered as two separate monomers. Further inspection of neighboring molecules in the crystal lattice also did not reveal any contacts other than typical crystal contacts. Based on its higher resolution and reduced complexity, only the structure of crystal form 2 was refined to completion and used for all subsequent discussions.

The crystals of apo TbrAK appeared to be single crystals but their diffraction was of very bad quality due to inherent defects that gave rise to many overlapping lattices. Despite extensive screening of crystals and handling conditions, we could not get interpretable data for the apo crystals. However, the diffraction from one of the apo crystals that had been soaked with compound **1**, while still exhibiting several overlapping lattices indicative of non-merohedral twinning, was of sufficient quality to allow indexing of a single lattice, yielding data of sufficient quality for model refinement to at resolution of 2.8 Å. Integration and scaling led to reasonable statistics considering the low I/sigma(I) (3.6 overall) of the data and the potential for overlap with other lattices. The crystal system was found to be tetragonal with space group P4_1_2_1_2 with one molecule in the asymmetric unit and without any contacts other than typical crystal contacts with neighboring molecules in the crystal lattice.

### Overall structure of TbrAK and its complexes

**Figure 1:**
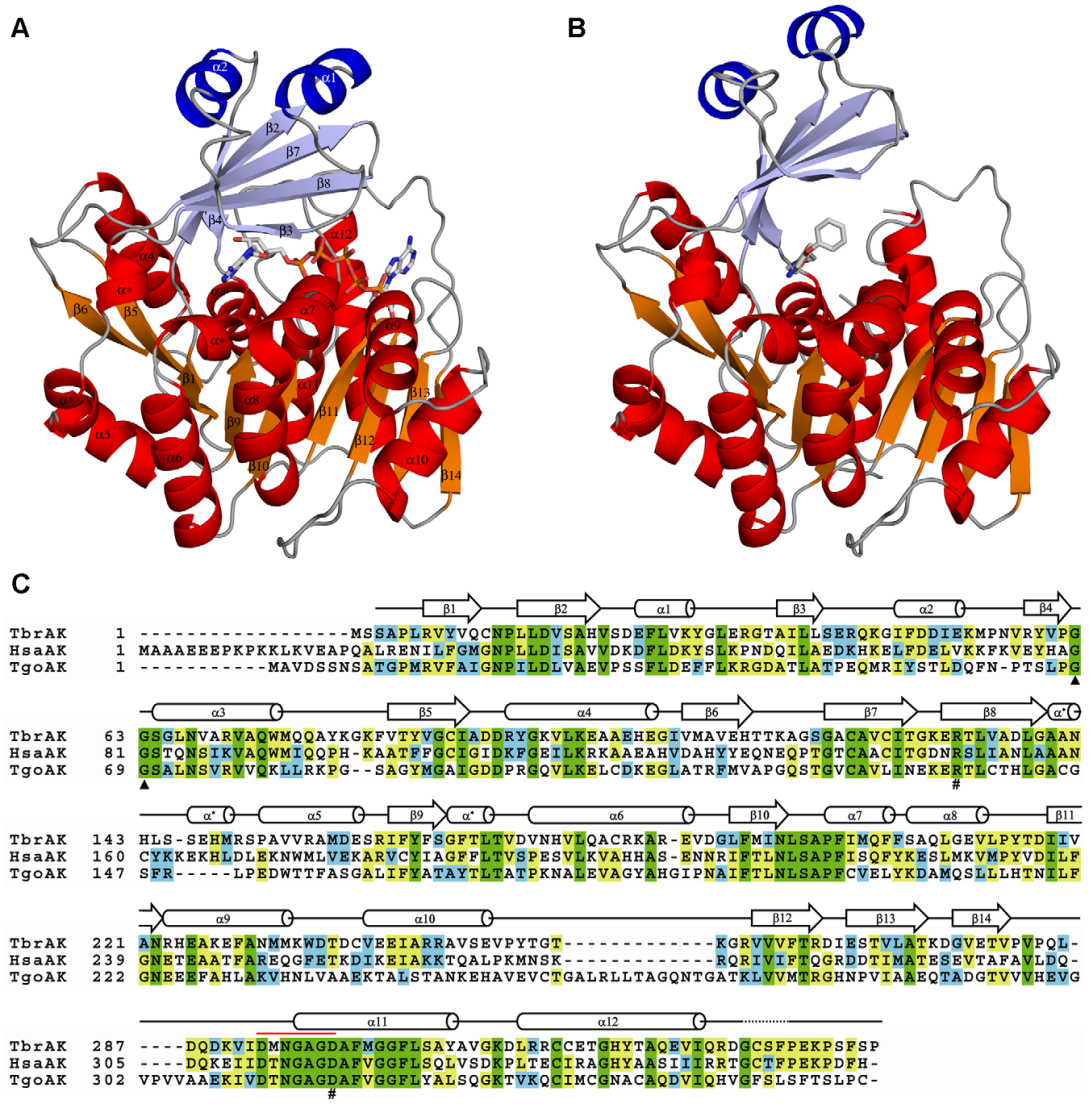
Ribbon diagram of TbrAK complexes and structural alignment. Panel A: Structure of TbrAK in complex with AP5A. Helices and β-sheets of the large domain are shown in red and orange, respectively while helices and β-sheets of the lid domain are shown in blue and light blue, respectively. Loops are depicted in gray, and AP5A is represented as stick model colored by atom type. Panel B: Overall structure of TbrAK complexed with compound **1**. The same coloring scheme is applied as in panel A. Panel C: Amino acid sequence alignment of TbrAK with HsaAK and TgoAK. Green indicates completely conserved residues, yellow indicates two or more highly conserved residues, and blue indicates at least one similar amino acid residue. The highly conserved di-glycine switch is marked with black triangles. The P-loop heptad (amino acids 293–299) is indicated by the red line. The catalytically important residues (R132 and D299) are marked with a pound (#) sign. The secondary structure elements of TbrAK, deduced from the TbrAK-AP5A structure, are schematically drawn above the amino acid sequences and depicted in panel A. As not all adenosine kinase structures exhibit 3/10 helices in the loop regions between β8/α5 and β9/α6, they are labeled with an asterisk (α*) in order to remain consistent with standard numbering applied to adenosine kinases. TbrAK exhibits 38% and 33% sequence identity compared to HsaAK and TgoAK, respectively.

The overall structures of both TbrAK in complex with AP5A (Figure 1, panel A) and compound **1** (Figure 1, panel B) contain a mixed fold and consist of 12 α-helices and 14 β-strands that form two distinct domains. The smaller lid domain appears as α/β two layer structure formed by a 5-stranded β-sheet (β2, β3, β4, β7, β8) and two solvent exposed α-helices (α1 and α2) located on top of it. The large domain is an α/β-domain built of a 9-stranded β-sheet (β1, β5, β6, β9-β14), of which only β13 is anti-parallel. The β-sheet is surrounded by 10 α-helices (α3-α12) exposed to the solvent (Figure 1, panel C). The overall structure of TbrAK is similar to recently described structures of adenosine kinases from human (HsaAK) [17], [18], *T. gondii* (TgoAK) [19], [20], [21], [22], and *M. tuberculosis* (MtuAK) [23], [24], although the sequence identity is moderate (HsaAK, sequence identity 38%; TgoAK, 33%; MtuAK, 17%). However, the most pronounced differences are found with MtuAK that acts as a dimer, and are located within the lid domain. MtuAK is comprised of two additional helices in the lid domain which acts as an interaction interface for creating the active MtuAK dimer while TbrAK, HsaAK and TgoAK appear exclusively as monomers. A comprehensive summary of all AK structures available in the PDB database with their corresponding conformational states, and the TbrAK structures presented here is given in Table S1.

**Figure 2:**
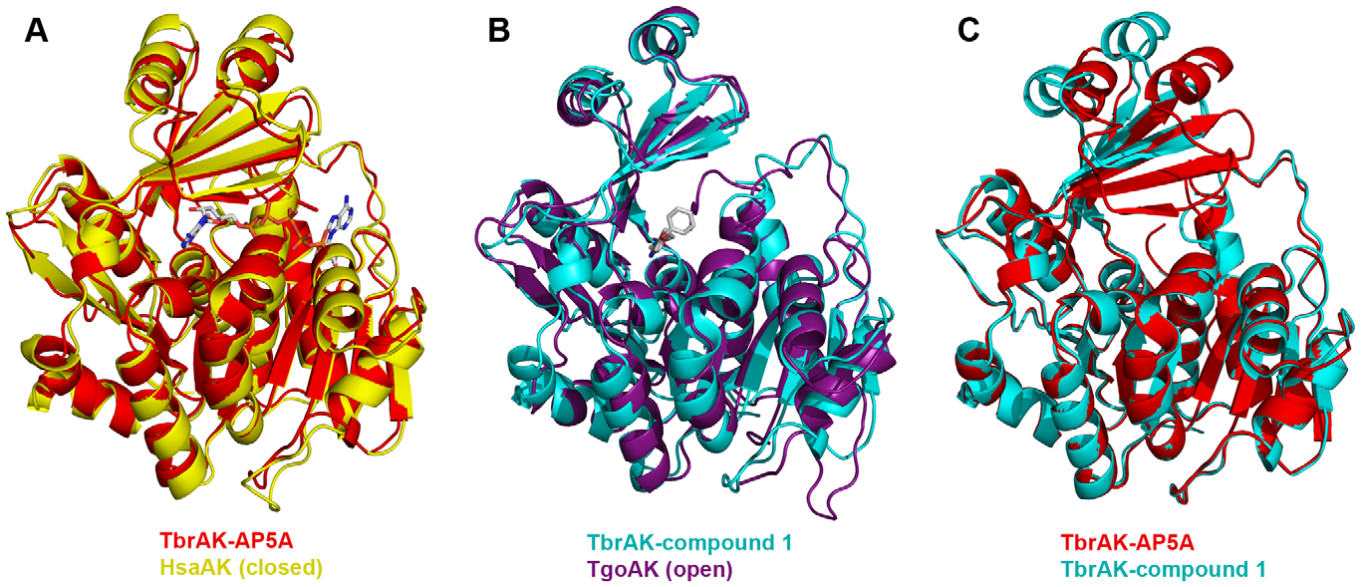
Comparison of TbrAK overall structures. Panel A: Superposition of TbrAK-AP5A (3OTX, shown in red) and HsaAK (1BX4, yellow) shows that TbrAK co-crystallized with the bisubstrate inhibitor adopts a closed conformation as found for HsaAK in complex with two adenosines (RMSD 0.99 Å). Panel B: In contrast, the complex of TbrAK and compound **1** (2XTB, cyan) adopts an open conformation and overlays very well with the apo structure of TgoAK (1LIO, purple; RMSD 1.20 Å). Panel C: The superposition of both TbrAK structures with respect to the large domain reveals a compact protein conformation for the TbrAK-AP5A complex (3OTX, red), while a more open conformation, by a rotation of around 30° of the small domain with respect to the large domain, can be observed for the TbrAK-compound **1** complex (2XTB, cyan).

Superposition of TbrAK with HsaAK and TgoAK clearly shows that TbrAK co-crystallized with the bisubstrate inhibitor AP5A adopts a closed conformation similar to the one observed in most adenosine kinase crystal structures where both binding pockets are occupied, e.g. TgoAK in complex with adenosine and the non-hydrolysable ATP analog AMP-PCP (PDB code 1LII [20]), and HsaAK in complex with two adenosines (PDB code 1BX4 [17] (RMSD 0.99 Å, Figure 2, panel A). This finding is not unexpected as AP5A mimics the two substrates adenosine and ATP involved in the enzymatic reaction. In contrast, the complex of TbrAK with compound **1** overlays very well (RMSD 1.20 Å) with the apo structure of TgoAK (1LIO) [20], thus exhibiting an open conformation (Figure 2, panel B). Indeed, most recent crystallographic studies have revealed that adenosine kinases undergo large conformational changes during substrate binding, and it was observed that the lid domain can adopt an open or a closed conformation, depending on the ligand bound. Apparently, similar structural transitions are possible and found for TbrAK (Figure 2, panel C).

### Adenosine and ATP binding site in TbrAK

**Figure 3:**
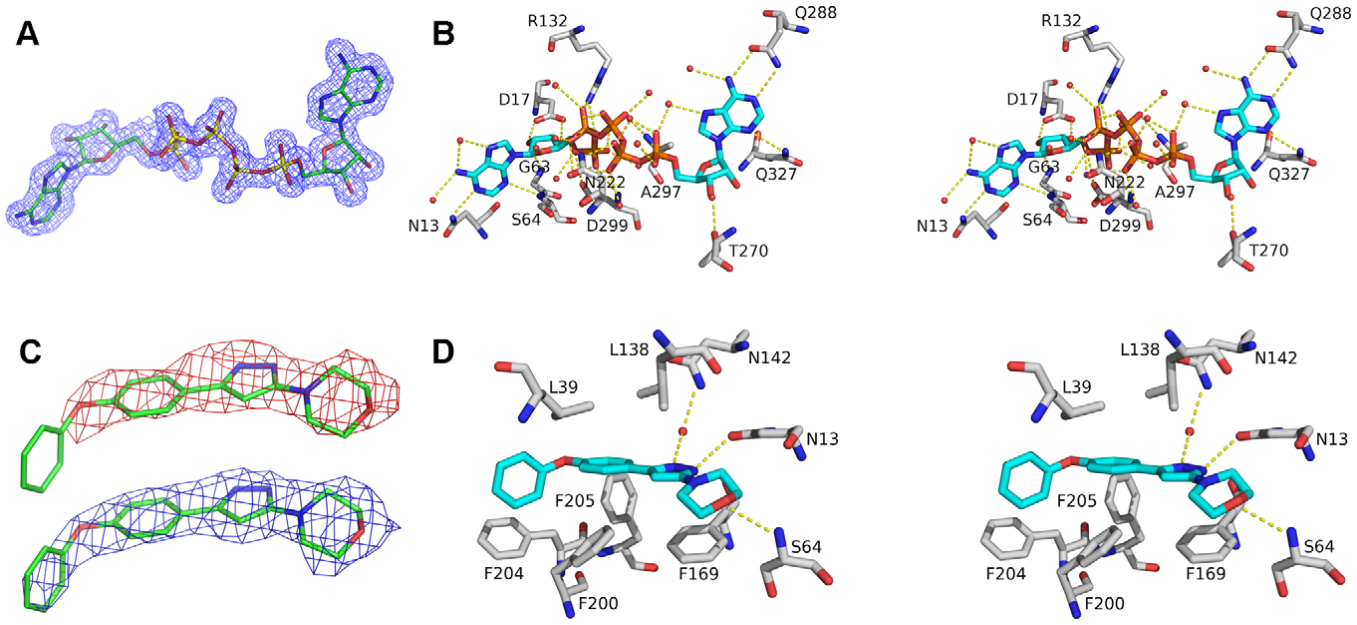
View on the binding site of both TbrAK complexes. Panel A: The electron density map of AP5A at 1.55 Å after refinement (2mFo-DFc), contoured at σ  =  1. Panel B: Stereo picture of the AP5A binding site. For the sake of clarity, only hydrophilic interactions are depicted. Amino acids are presented as sticks with carbon atoms colored in gray. Oxygen atoms are red, nitrogen atoms blue, and phosphor atoms orange. Carbon atoms of AP5A are shown in blue. Hydrogen bonds are shown as yellow dotted lines, and water molecules forming water-mediated hydrogen bonds are indicated as red spheres. For the sake of clarity further amino acids interacting with water molecules were omitted. Panel C: The electron density around compound **1** calculated at 2.8 Å resolution before its inclusion in the model (mFo-DFc, contoured at σ  =  1, map colored in red), and after refinement with compound **1** (2mFo-DFc, contoured at σ  =  1, map shown in blue). Panel D: Stereo picture of the compound **1** binding site. The same color scheme is applied as in panel B.

The bisubstrate inhibitor AP5A was co-crystallized with TbrAK which adopts the closed conformation in presence of this compound. The electron density of AP5A was clearly resolved in the structure and provides a detailed view into the adenosine and the ATP binding site (Figure 3, panel A). AP5A exhibits an extensive hydrogen bonding pattern with the protein either through direct or water-mediated interactions (Figure 3, panel B).

The interactions in the adenosine binding pocket are mainly water-mediated, apart from the side chain NH_2_ group of N13 which makes a hydrogen bond with nitrogen N1 of adenosine and the side chain of D17 which forms bidentate interactions with the two OH-groups of the ribose. The side chains of L15, L39, V125, C123, L134, L138 and F169 are involved in hydrophobic contacts with the adenine moiety in the adenosine binding site. The phosphate groups of AP5A are stabilized by water mediated interactions and by the side chain NH_2_ groups of R132 and N222 and the backbone nitrogen of A297 (Figure 3, panel B).

The adenosine part of AP5A in the ATP-binding pocket shows hydrogen bonds to the side chains of Q288 (to the free NH_2_ group of adenine) and Q327 (to a nitrogen atom of the pyrimidine ring of adenine), and the ribose moiety is stabilized by backbone interactions with D266 and T270. Hydrophobic contacts with the adenosine moiety in the ATP binding pocket are formed by I267, V283, L286, V291, F301, H323 and I330 (Figure 3, panel B). All other interactions with the bisubstrate inhibitor in this pocket are water-mediated involving residues N13, I38, S64, F169, T172, D299 (adenosine binding pocket), R132, N222, T264, G296, A297 (pentaphosphate moiety) and R265, E268, T270 (ATP binding pocket). The residues in the adenosine binding pocket directly involved in polar interactions with the adenosine moiety are completely conserved between HsaAK and TbrAK, while residues forming the ATP binding pocket are less conserved. Residue D266 in TbrAK is replaced by a glycine in HsaAK, however only the backbone of this residue is involved in polar interactions with the adenosine moiety of AP5A.

### The binding site of compound 1 in TbrAK

As outlined above, TbrAK in complex with compound **1** adopts an open conformation, and the location of compound **1** was unambiguously identified by its well-defined electron density (Figure 3, panel C) localized in part in the adenosine binding site in the adenine moiety binding area, but pointing away from the ribose binding region towards the solvent. The elongated shape of the compound initially suggested two possible orientations in the mFo-DFc and the 2mFo-DFc density maps: with the morpholine moiety being solvent exposed (see Figure S1, panel A) or, after a rotation by 180°, pointing toward the protein (Figure S1, panel B). However, the first binding mode could be excluded as the two phenyl rings of the phenoxyphenyl moiety would need to be coplanar in order to fit the flat density. This conformation is sterically inaccessible as shown by large clashes in the MolProbity [39] analysis (Figure S2, panel A). In contrast, compound **1** fits very well into the observed density with only minor clashes (Figure S2, panel B) between the morpholine and the pyrazole moiety. Moreover, only this orientation satisfies a number of hydrogen bonds to the protein (Figure 3, panel D; Figure S1, panel C), supporting the conclusion that compound **1** binds to TbrAK with the phenoxyphenyl moiety pointing towards the outside and the morpholine being buried in the active site.

Compound **1** forms three hydrogen bonds with the protein, namely with the side chain NH_2_ groups of N13 and N142 (water-mediated) and the main chain nitrogen of S64. The phenoxy moiety of compound **1** interacts mainly via hydrophobic contacts formed by F169, F200, F204 and F205 (Figure 3, panel D). The observed binding mode is very similar to the one found for an alkynylpyrimidine inhibitor bound to HsaAK (2I6B) [18]. Moreover, in presence of this inhibitor the lid domain adopts the open conformation. However, despite their overlapping binding modes, compound **1** is an activator of TbrAK while the alkynylpyrimidine inhibits the enzyme (see below for a detailed discussion).

### The TbrAK structures and implication on the hyperactivation mechanism

The recent crystallographic studies of adenosine kinases in presence and absence of substrates revealed the catalytic mechanism. It is assumed that adenosine binds first to the adenosine binding site, thereby inducing a 30° hinge bending motion that closes the lid domain to a pre-catalytic conformation by means of the highly conserved Gly-Gly (G62 and G63; TbrAK numbering, see Figure 1, panel C) conformational switch. Once the conformational change has occurred, subsequent ATP binding induces additional local structural changes via the adenine moiety and the β and γ phosphates, creating an extensive anion hole formed by the P-loop heptad DMNGAGD located at the N-terminal part of helix α11 (amino acids 293–299, see Figure 1, panel C). Once the structure is completely closed, adenosine and the γ phosphate of ATP are entirely sequestered from the solvent and the direct phosphate transfer from ATP to adenosine can take place. Moreover, it was found that substrate inhibition, which is a common property of adenosine kinases including TbrAK [10], [15], occurs by competitive binding of adenosine to the ATP binding site.

**Figure 4:**
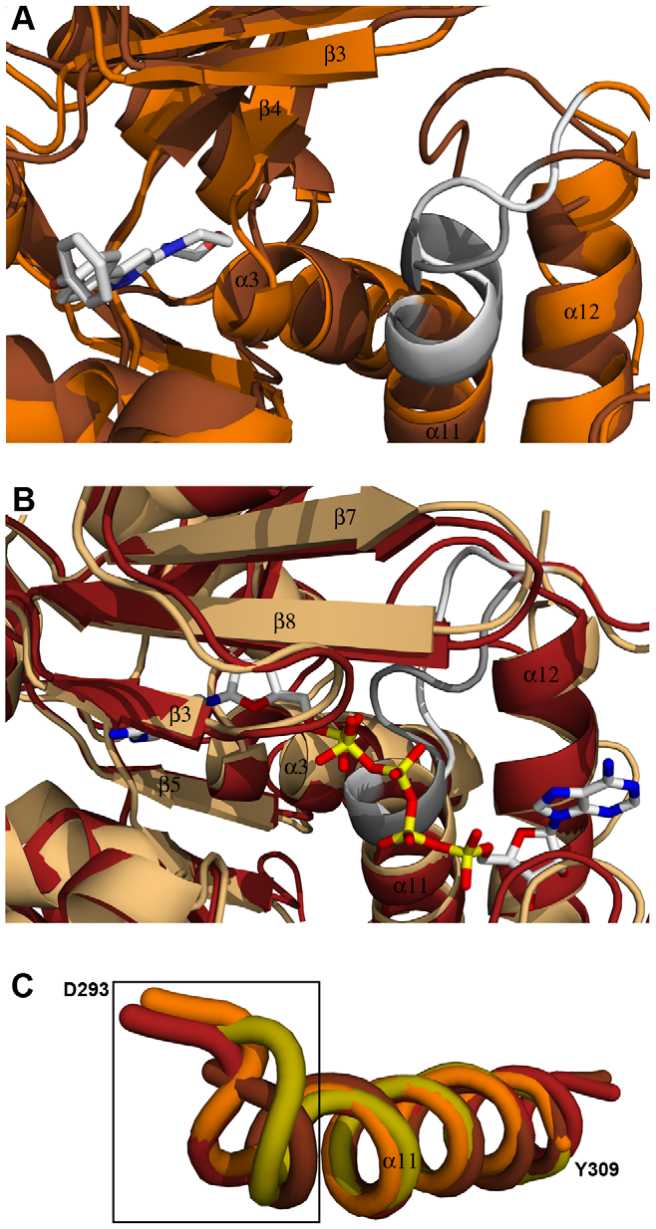
Anion hole formation in TbrAK. Panel A: Superposition of apo TgoAK (1LIO, open conformation, brown) and the complex of TbrAK and compound **1** (2XTB, open conformation, orange) showing the area involved in the formation of the anion hole. The P-loop heptad (D293-D299) forming the anion hole is shown in white for TbrAK-compound **1** and in gray for TgoAK. Although exhibiting an open conformation, the P-loop heptad in the TbrAK-compound **1** complex is not integrated anymore in helix α11. Panel B: Superposition of TgoAK in complex with adenosine and AMP-PCP (1LII, closed conformation, rust-red) and TbrAK-AP5A (3OTX, closed conformation, light orange) focusing on the area of the anion hole. The P-loop heptad is shown in white for TbrAK-AP5A and in gray for TgoAK. Both P-loop heptads are integrated in helix α11, however, the heptad in TbrAK-AP5A is distorted, most likely due to the presence of the additional phophates in AP5A. Panel C: Superposition and close-up view on helix α11 and the corresponding P-loop heptad of selected adenosine kinases. For the sake of clarity only the first amino acid of the TbrAK heptad (D293) and the last amino acid of helix α11 in TbrAK (Y309) are labeled. The selected structures are TbrAK-compound **1** (2XTB, open conformation, orange), TgoAK in complex with adenosine and AMP-PCP (1LII, closed conformation, rust-red), the apo TgoAK (1LIO, open conformation, brown), and the structure of HsaAK in complex with the alkynylpyrimidine inhibitor (2I6B, open conformation, olive). The black square indicates the area of anion formation at the N-terminus of helix α11. With the exception of TbrAK-compound **1**, the structures exhibiting an open conformation have their P-loop heptads integrated in helix α11, while upon substrate binding the first turn of helix α11 unfolds to form the anion hole.

Typically, ATP induces the formation of the anion hole which is created via a helix to coil transition to stabilize the negative charge of the phosphate residues. Surprisingly, although the lid domain in the structure of TbrAK with compound **1** adopts the open conformation, the anion hole is identical to the anion hole found in adenosine kinases adopting the closed conformation like in TgoAK in complex with adenosine and the non-hydrolysable ATP analog AMP-PCP (1LII) [20]. For comparison, the apo form of TgoAK (1LIO) does not form the anion hole due to the absence of ATP, which can be seen by the fact that the anion hole motive DTNGAGD (312–318) in TgoAK is still integrated in the α11 helix and therefore not able to stabilize negative charges of phosphate residues (Figure 4, panels A and C). Thus compound **1** is able to induce the formation of the anion hole also in absence of ATP, which could favor ATP rather than adenosine binding in the ATP binding pocket due to favorable interactions with the β and γ phosphate groups. As a result, the intrinsic substrate inhibition would be abolished, which corresponds exactly to the recent observation of abolishment of substrate inhibition conferred by compound **1** towards TbrAK [15]. Interestingly, the anion hole formed is largely distorted in the TbrAK-AP5A complex when compared to the closed conformations of other adenosine kinases (e.g. TgoAK in 1LII), which may be explained by the two additional phosphate groups linking the two adenosine moieties of AP5A (Figure 4, panel B).

The above findings give rise to the question of how the catalytic reaction can still take place with compound **1** occupying part the adenosine binding pocket. There are several possible answers including that adenosine replaces compound **1** in the adenosine binding site after ATP binding (ATP binding prior to adenosine binding), or that adenosine joins compound **1** in the adenosine binding site thereby widening the adenosine binding pocket in such a way that the two compounds stabilize each other by aromatic stacking interactions, or that compound **1** is forced to move to a second lower affinity binding site while adenosine could occupy the adenosine binding site. Indeed, a recent ITC study has revealed two binding sites for compound **1** on TbrAK [15].

### TbrAK complex stability analyzed by the thermal denaturation assay and ITC

In order to better understand the binding mechanism of compound **1** and TbrAK substrates, we analyzed adenosine and ATP binding in absence and presence of the activator. The presence of a ligand stabilizes a protein conformation, which results in increased thermal stability reported as a higher melting point (T_m_) for the complex compared to the apo structure [40]. As shown in Table 2, apo TbrAK melted at 43.1±0.4°C while the maximum stability was achieved either by adenosine alone (ΔT_m_ 7.6°C) or by adenosine and ATP (ΔT_m_ 7.4°C). Interestingly, ATP combined with adenosine seems not to increase further the T_m_ of apo TbrAK which is in agreement with the fact that ATP alone does not shift T_m_ of apo TbrAK (ΔT_m_ −0.3°C). This corresponds very well to the observation that adenosine triggers structural conformations that initiate the formation of the ATP binding site, and is in agreement with the suggested ordered bi-bi mechanism of substrates binding involved in the enzymatic reaction [20]. Interestingly, the bisubstrate inhibitor AP5A also increased T_m_ (ΔT_m_ 7.4°C) at the same level as adenosine and adenosine/ATP, reflecting the fact that AP5A binds to both binding sites at the same time.

**Table 2:** Thermal stability assay regarding TbrAK in absence and presence of AP5A, adenosine, ATP and compound 1.

	T_m_ [°C] ± SD^a^	Δ T_m_ [°C]
TbrAK	43.1±0.4	−
TbrAK + compound **1**	47.9±0.1	4.8
TbrAK + AP5A	50.5±0.2	7.4
TbrAK + AP5A + compound **1**	50.1±0.1	7.0
TbrAK + adenosine	50.7±0.2	7.6
TbrAK + adenosine + compound **1**	51.1±0.1	8.0
TbrAK + ATP	42.8±0.1	−0.3
TbrAK + ATP + compound **1**	47.0±0.1	3.9
TbrAK + adenosine + ATP	50.5±0.1	7.4
TbrAK + adenosine + ATP + compound **1**	51.2±0.1	8.1

a Values represent the average of three experiments.

Compound **1** alone increased the thermal stability (ΔT_m_ 4.8°C) compared to apo TbrAK, whereas the combination of compound **1** and ATP did not further improve thermal stability (ΔT_m_ of 3.9°C) compared to compound **1** alone. The melting point of TbrAK in presence of adenosine or both adenosine and ATP was slightly increased by 0.4°C and 0.7°C, respectively, when compound **1** was added.

**Figure 5:**
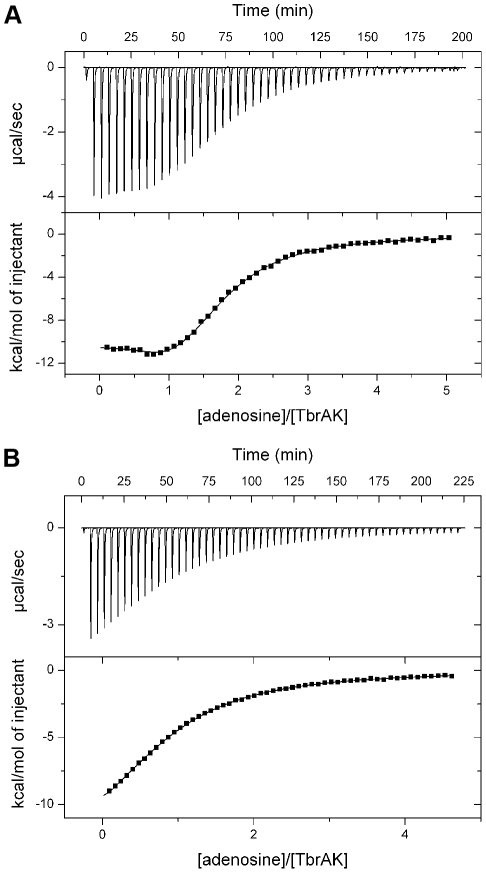
Binding of adenosine to TbrAK measured by ITC. Panel A: The top panel shows a representative ITC experiment for the injection of adenosine (2.17 mM) into the sample cell containing TbrAK (87 µM). The binding isotherm obtained by integration and normalization of the raw data and by correction for the heat of ligand dilution is shown on the lower panel. The solid line represents the non-linear least square fit based on a two-sites non-interacting binding model. As indicated by the molar ratio two molecules of adenosine bind to TbrAK, one via a high affinity binding site with a K_D_ of 1.3±0.3 µM and a ΔH_bind_ of −11.1±0.7 kcal/mol, and one via a low affinity site exhibiting a K_D_ of 27.6±1.1 µM and a ΔH_bind_ of −17.9±0.3 kcal/mol (n_1_ and n_2_ equaling 0.9±0.1 and 0.8±0.1, respectively). Panel B: The binding isotherm obtained by the titration of adenosine (2.0 mM) into TbrAK (90 µM) in presence of compound **1** (150 µM) was fit applying a single-site binding model. One molecule of adenosine binds to the enzyme with a K_D_ of 53.2±0.8 µM and a ΔH_bind_ of −15.5±0.2 kcal/mol (n  =  0.9±0.1). The mean of two independent experiments is reported.

Further binding experiments of adenosine toward TbrAK by means of ITC revealed that adenosine binds to two binding sites (Figure 5, panel A), yielding a high affinity binding site with a K_D_ of 1.3±0.3 µM and a ΔH_bind_ of −11.05±0.65 kcal/mol, while the low affinity site exhibited a K_D_ of 27.6±1.1 µM and ΔH_bind_ of −16.41±0.33 kcal/mol. This is in agreement with recent crystal structures showing two adenosines bound to adenosine kinase [17], [20]. It is likely that the high affinity site corresponds to the adenosine binding site (adenosine as substrate) while the second adenosine would bind to the ATP binding site with lower affinity, thus leading to the intrinsic substrate inhibition due to competitive inhibition of ATP binding as observed in TbrAK [15] and other adenosine kinases [11], [12], [13], [14]. When carrying out the experiment in presence of compound **1**, adenosine changes its binding properties (Figure 5, panel B). Only a single binding site can be observed, and the K_D_ is found to be 53.2±0.8 µM (ΔH_bind_  =  −15.5±0.2 kcal/mol). Due to the fact that the formation of the anion hole alone would not be sufficient to completely abolish adenosine binding at the ATP binding site, and that the adenosine binding site is occupied by the high affinity activator, the observed single and increased K_D_ is likely to present adenosine binding to the ATP binding site, in particular in absence of competing ATP. This finding supports the hypothesis that the release of compound **1** and the subsequent substrate binding at the adenosine binding site may first require ATP binding at the ATP binding site as suggested above.

### Structure-activity relationships of compound 1 and derivatives

**Figure 6:**
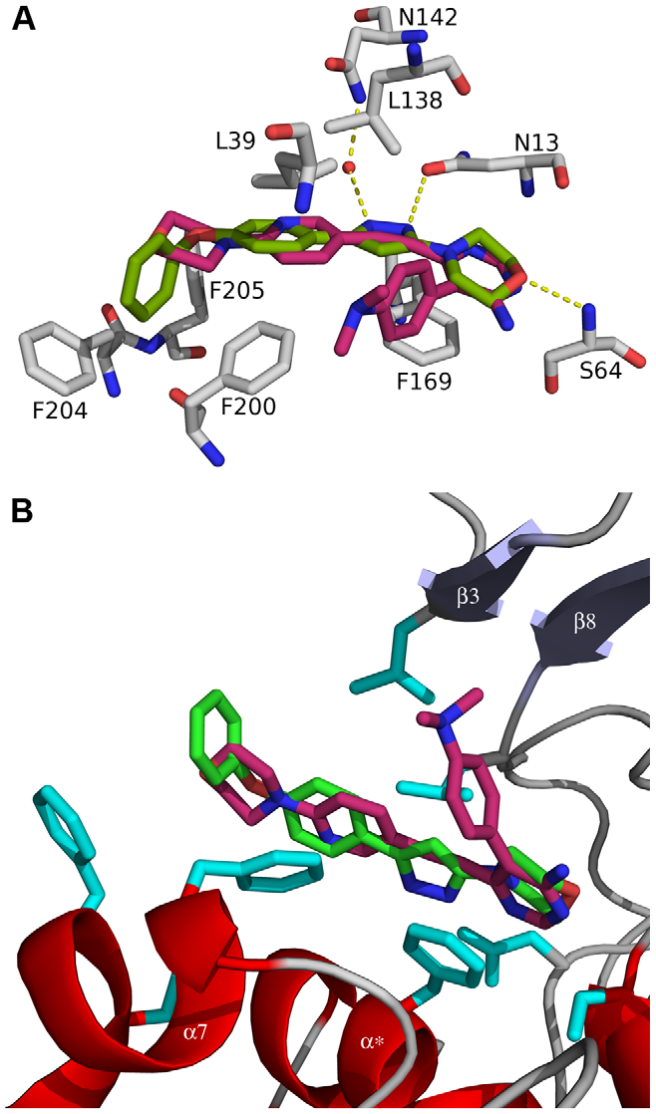
Binding mode of compound 1 in TbrAK and of the alkynylpyrimidine inhibitor in HsaAK. Panel A: Superposition of HsaAK (2I6B) and TbrAK (2XTB) shows that the alkynylpyrimidine inhibitor (pink) binds in a very similar orientation to HsaAK as compound **1** (green) to TbrAK. Panel B: The same superposition showing the binding modes of activator and inhibitor in context of TbrAK. The dimethylaminophenyl moiety of the inhibitor points towards strand β3 of the lid domain (light blue), thus stabilizes the open conformation by hindering the domain closure movement. The residues colored in cyan are involved in compound **1** binding and are the same as presented in panel A. For the sake of clarity only the TbrAK chain is shown, and parts of the loop connecting β8 and α5 (including N142) have been omitted.

A very interesting aspect of TbrAK in complex with compound **1** is the fact that the binding mode is very similar to the one of the alkynylpyrimidine inhibitor in HsaAK (2I6B) [18], and in both structures the lid domain remains in the open conformation (Figure 6, panel A). However, compound **1** is an activator of TbrAK while the alkynylpyrimidine inhibits the enzyme despite the overlapping binding mode. On the one hand, these different modes of action could be explained by the fact that the alkynylpyrimidine inhibitor is not able to induce the anion hole (Figure 4, panel C) needed for abolishment of substrate inhibition and for the improved accommodation of ATP. On the other hand, the dimethylaminophenyl moiety of the alkynylpyrimidine inhibitor, which has no overlapping counterpart in compound **1**, is strongly interacting with the lid domain stabilizing its open conformation of the adenosine kinase and thus acts as an inhibitor of the enzyme (Figure 6, panel B). As compound **1** is not strongly interacting with the lid domain, it is most probably not able to interfere with the hinge bending motion to close the lid domain.

**Figure 7:**
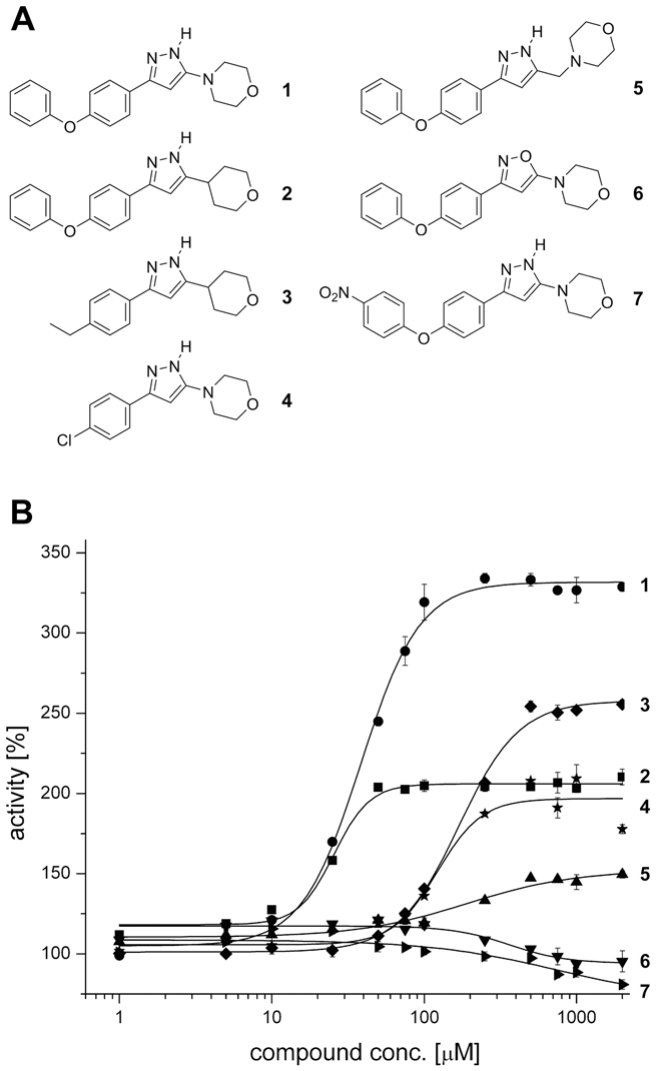
Structure and activity of phenoxyphenyl-pyrazoles. Panel A: Structure of the title compound 4-[5-(4-phenoxyphenyl)-2*H*-pyrazol-3-yl]morpholine (compound **1**) and its derivatives 3-(4-phenoxyphenyl)-5-(tetrahydropyran-4-yl)-1*H*-pyrazole (**2**), 3-(4-ethylphenyl)-5-(tetrahydropyran-4-yl)-1*H*-pyrazole (**3**), 4-[5-(4-chlorophenyl)-2*H*-pyrazol-3-yl]morpholine (**4**), 4-[5-(4-phenoxyphenyl)-2*H*-pyrazol-3-ylmethyl]morpholine (**5**), 4-[3-(4-phenoxyphenyl)isoxazol-5-yl]morpholine (**6**), and 4-{5-[4-(4-nitrophenoxy)phenyl]-2*H*-pyrazol-3-yl}morpholine (**7**). Panel B: Concentration dependence of the activation effect on TbrAK of compounds **1** to **7** in the range of 1–2000 µM. Increasing concentrations of compound **1** (black circle) gives a sigmoid saturation curve for TbrAK activation, yielding an EC_50_ value of 38.9±0.9 µM. Similar sigmoid saturations showing their potency are observed for compound **2** (black square), **3** (black diamond), **4** (black star), and **5** (black triangle), yielding in EC_50_ values of 25.4±0.2 µM (**2**), 168.4±1.1 µM (**3**), 134.7±11.6 µM (**4**), and 195.4±6.0 µM (**5**), respectively. Values represent the average of three independent experiments.

Besides a lack of interaction with the lid domain, however, above findings raise the question regarding the structural elements making compound **1** work as an activator of TbrAK. To address this issue, we have analyzed the potency and efficacy of a series of derivatives [29]. The activation conferred by compound **1** and its derivatives (compounds **2**–**7**, Figure 7, panel A) is illustrated in Figure 7 (panel B), and corresponding values are summarized in Table 3. Compound **2** that consists of a tetrahydropyran ring instead of the morpholine ring in the original compound **1**, is still able to activate TbrAK at similar potency (EC_50_ 25.4±0.2 µM) but at lower efficacy (2.0 fold) compared to compound **1** (EC_50_ 38.9±0.9 µM and efficacy 3.5 fold, respectively). Replacing the phenoxy moiety of compound **2** by an ethyl group (compound **3**), leads to a significantly decreased activation potency (EC_50_ 168.4±1.1 µM), while efficacy remains 2.5 fold. Compound **4,** which harbors an intact morpholine/pyrazole moiety and has the phenoxy moiety substituted with a chlorine atom, still performs with an EC_50_ of 134.7±11.6 µM and an efficacy of 2.0 fold. An additional methylene group between the morpholine and pyrazole moiety (compound **5**) lowers the potency substantially (EC_50_ of 195.4±6.0 µM), while activation efficacy is reduced to 1.5 fold. The replacement of the pyrazole by an isoxazol ring (compound **6**) or substitution of the phenoxy moiety by a nitro group in para position (compound **7**) loses all activation capacity. Taken together, these results indicate the importance of an accessible morpholine and pyrazole moiety in immediate coexistence, and a non-substituted phenoxyphenyl moiety for hyperactivation of TbrAK.

**Table 3:** Activity data with respect to TbrAK inhibition by AP5A and activation by compounds 1–7.

	potency^a^	efficacy increase^b^
TbrAK + AP5A	IC_50_ 29.4±0.2 µM	
TbrAK + compound **1**	EC_50_ 38.9±0.9 µM	3.5 fold
TbrAK + compound **2**	EC_50_ 25.4±0.2 µM	2.0 fold
TbrAK + compound **3**	EC_50_ 168.4±1.1 µM	2.5 fold
TbrAK + compound **4**	EC_50_ 134.7±11.6 µM	2.0 fold
TbrAK + compound **5**	EC_50_ 195.4±6.0 µM	1.5 fold
TbrAK + compound **6**	n.e.^c^	n.e.^c^
TbrAK + compound **7**	n.e.	n.e.^c^

a Values represent the average of three experiments.

b The efficacy of TbrAK in absence of compound was taken as reference.

c no effect, i.e. compounds neither act as activator nor as inhibitor.

The investigation of the specific interactions of compound **1** in the active site enables a consistent structure activity relationship. As shown in Figure 3 (panel B) compound **1** makes a direct hydrogen bond with the oxygen of its morpholine moiety to the backbone nitrogen of S64, a direct hydrogen bond with nitrogen N1 of the pyrazole ring to N13, and a water-mediated hydrogen bond with nitrogen N2 of the pyrazole ring and the side chain of N142. Due to low resolution it is not known which tautomer conformation of the pyrazole ring is present, but the tautomers may induce a switch of the side chain amide of N13 and perhaps N142 to put the electron acceptors and donors in the correct positional arrangement. Both possible tautomers are conceivable, however, experimental results and the SAR outlined strongly suggest that the NH of the pyrazole ring is pointing toward the oxygen atom of N13, resulting in a hydrogen bond interaction. In addition, there are hydrophobic interactions of the morpholine moiety with the parallel situated F169 and the terminal phenyl ring with the hydrophobic patch formed by F200, F204, and F205 at the surface of TbrAK.

Compound **2** exhibits similar potency but is less efficient than compound **1**. The only difference between both activators is that in compound **2** the nitrogen of the morpholine ring is replaced by a carbon atom. While the carbon atom will maintain the chair conformation, it may have a significant influence on the pK_a_ value of nitrogen N1 in the pyrazole moiety by rendering it more acidic due to smaller electronegativity of a carbon atom. The subsequent reduction of the hydrogen bond strength may account for the loss of efficacy with respect to TbrAK activation. A similar effect may explain the low activation efficacy of compound **5** where a methylene group separates the pyrazole and the morpholine moiety. In addition, the methylene group most likely disrupts the hydrogen bond pattern of the pyrazole and could thus lead to the observed unfavorable effect on both potency and efficacy of the compound. For compounds **3** and **4** the situation is more complex as, at similar potency, the tetrahydropyran ring in compound **3** leads to higher efficacy than the morpholine ring in compound **4**. However, their loss of the phenoxy moiety and replacing it by an ethyl group and a chlorine atom, respectively, makes it difficult to estimate their influence on the acidity of N1 in the pyrazole ring. The loss of activation capacity observed for compound **6** can be explained by the exchange of the pyrazole with an isoxazole ring. The oxygen of the isoxazol ring leads to a repulsive oxygen-oxygen interaction and eliminates the favorable hydrogen bond interaction found experimentally for compound **1**. This supports the presence of the tautomer form and the hydrogen bond pattern depicted in the crystal structure (Figure 3, panel D). Interestingly, compound **7** loses activity completely due to the para-substitution on the phenoxyphenyl moiety. With the nitro group pointing to the solvent, steric hindrance can be excluded as the source of this observation. However, the strong electron-withdrawing substituent in para-position will change the electrostatic potential on the surface of the phenoxy-moiety [41] which may induce repulsive interactions with respect to the hydrophobic patch formed by F200, F204, and F205, possibly leading to unfavorable conformational changes that interfere with binding.

Based on the structural and biochemical data we conclude that potency is related to interaction of the compounds within the binding site via morpholine, pyrazole and phenoxy moiety, with the consequence that the interaction with N13 may be directly involved in more or less pronounced anion hole formation. Interestingly, both hydrogen bonding partners of compound **1**, amino acids N13 and N142, are located within two flexible linkers connecting the small lid domain with the large domain. Considering that domain closing upon adenosine binding during the catalytic cycle may initiate the formation of the anion hole via N13 and N142 and long range effects, we may assume that compound **1** while binding to the active site and forming strong hydrogen bonds to N13 and N142 could trigger anion hole formation without domain closure as shown by the crystal structure.

### Conclusion

The present study reports the first three-dimensional crystal structures of *T. b. rhodesiense* adenosine kinase, an important enzyme in the parasite purine salvage pathway, in complex with the bisubstrate inhibitor AP5A (1.55 Å resolution), and in complex with the recently discovered activator 4-[5-(4-phenoxyphenyl)-2*H*-pyrazol-3-yl]morpholine (compound **1**) at 2.8 Å resolution. The subsequent structural analysis sheds light on substrate and activator binding, and very importantly, gives insight into the possible mechanism of the abolishment of the intrinsic substrate inhibition in presence of compound **1** [15] that leads to hyperactivation. The ultimate link between hyperactivation and cell toxicity is currently under investigation.

The structure-activity relationships in terms of TbrAK activation properties support the observed binding mode of compound **1** in the crystal structure and give hints about anion hole formation independent of domain closure. These results may open the field for subsequent optimization of this compound series with respect to pharmacodynamics and pharmacokinetics, which will be essential for developing this novel trypanocidal strategy.

## Supporting Information

Figure S1 **Binding mode analysis of compound 1.** While the location of compound **1** was unambiguously identified by its well-defined electron density, the nature of the compound suggests two possible orientations. Panel A: Stereo picture of the 2mFo-DFc map calculated from the model before including compound **1** in the refinement showing compound **1** modeled with the morpholine moiety exposed to the solvent. In order for the compound to fit into the flat density, the two phenyl rings of the phenoxyphenyl moiety need to be in a sterically inaccessible coplanar conformation. Panel B: The same view with compound **1** rotated by 180° and fit into the flat density. In this orientation the morpholine moiety points toward the protein without adopting sterically unfavorable conformations within the phenoxyphenyl moiety. Panel C: The same model as in B with the 2mFo-DFc map calculated from the final model including compound **1** and showing the distinct hydrogen bonding pattern which is only possible with compound **1** bound in this orientation. The panels show amino acids that are within 5 Å of compound **1**, with the addition of N142 as it is involved in the hydrogen bonding network with the bridging water. All maps are contoured at σ  = 1.
(PDF)

Figure S2 **MolProbity analysis of two possible binding modes of compound 1.** Panel A: When compound **1** is bound to TbrAK with the morpholine moiety pointing toward the solvent, the two phenyl rings of the phenoxyphenyl moiety need to be coplanar in order to fit the flat density. However, this conformation is sterically inaccessible as shown by large clashes in the MolProbity analysis (red and pink, Clashscore of 69.77) [1], which excludes this binding mode. Panel B: In contrast, compound **1** bound to TbrAK with the phenoxyphenyl moiety pointing towards the solvent while exhibiting a sterically favorable conformation, only minor clashes (yellow and orange, Clashscore of 23.26) between the morpholine and the pyrazole moiety are observed. This supports the conclusion that compound **1** binds to TbrAK with the phenoxyphenyl moiety pointing towards the outside and the morpholine being buried in the active site.
(PDF)

Table S1 **A comprehensive summary of all adenosine kinase structures published as of 2010, including the TbrAK structures presented in this work.**
(PDF)
