# Supplementary material for: Crystal Structures of T. b. rhodesiense Adenosine Kinase Complexed with Inhibitor and Activator: Implications for Catalysis and Hyperactivation
Source: PLoS Negl Trop Dis. 2011 May 24;5(5):e1164. doi: 10.1371/journal.pntd.0001164 (PMC3101181; doi:10.1371/journal.pntd.0001164)
Supplement: Figure S1 — Binding mode analysis of compound 1. While the location of compound 1 was unambiguously identified by its well-defined electron density, the nature of the compound suggests two possible orientations. Panel A: Stereo picture of the 2mFo-DFc map calculated from the model before including compound 1 in the refinement showing compound 1 modeled with the morpholine moiety exposed to the solvent. In order for the compound to fit into the flat density, the two phenyl rings of the phenoxyphenyl moiety need to be in a sterically inaccessible coplanar conformation. Panel B: The same view with compound 1 rotated by 180° and fit into the flat density. In this orientation the morpholine moiety points toward the protein without adopting sterically unfavorable conformations within the phenoxyphenyl moiety. Panel C: The same model as in B with the 2mFo-DFc map calculated from the final model including compound 1 and showing the distinct hydrogen bonding pattern which is only possible with compound 1 bound in this orientation. The panels show amino acids that are within 5 Å of compound 1, with the addition of N142 as it is involved in the hydrogen bonding network with the bridging water. All maps are contoured at σ = 1. (PDF) [file pntd.0001164.s001.pdf]

Supporting Information Figure S1

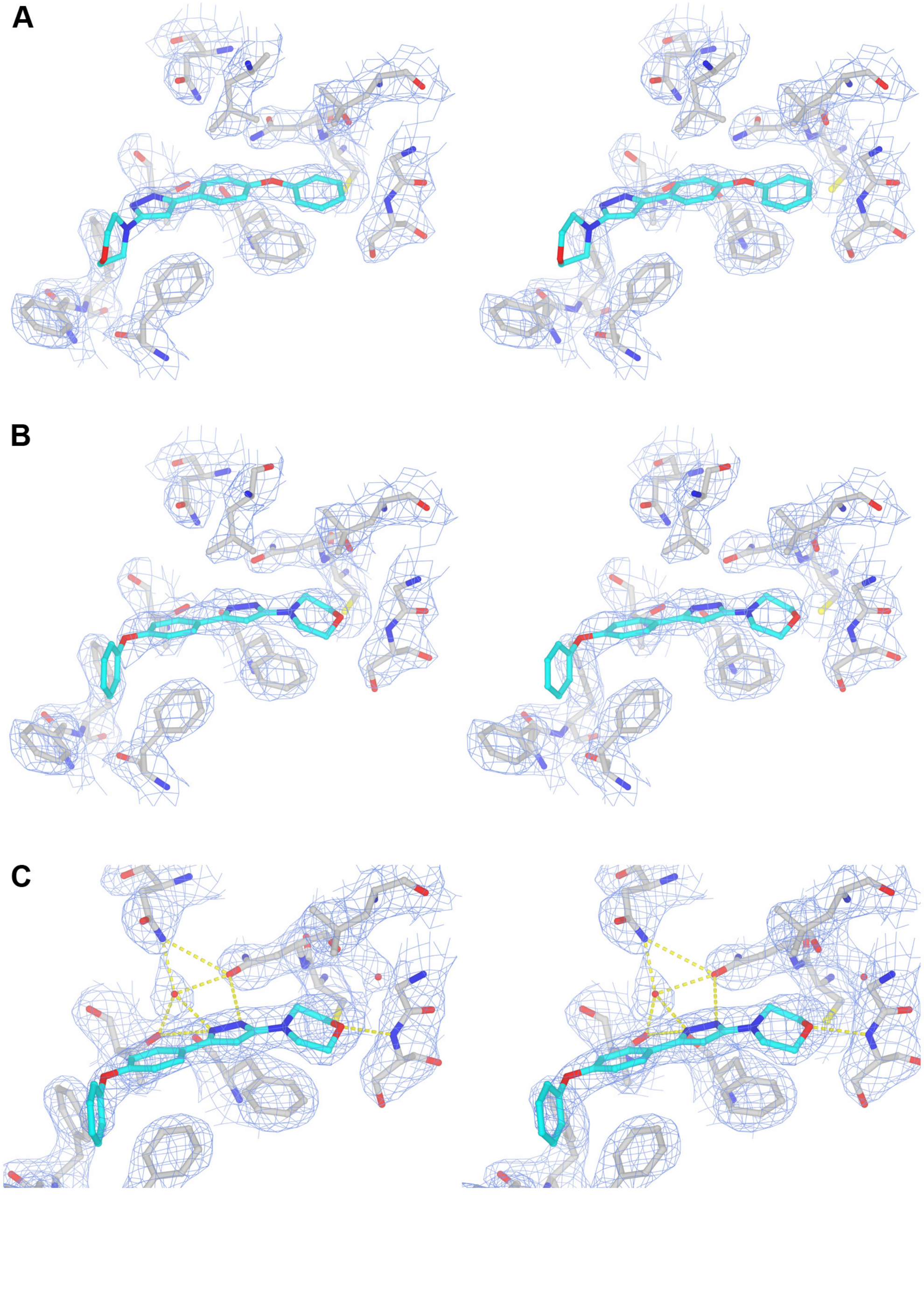

Figure S1. Binding mode analysis of compound 1. While the location of compound **1** was unambiguously identified by its well-defined electron density, the nature of the compound suggests two possible orientations. Panel A: Stereo picture of the 2mFo-DFc map calculated from the model before including compound **1** in the refinement showing compound **1** modeled with the morpholine moiety exposed to the solvent. In order for the compound to fit into the flat density, the two phenyl rings of the phenoxyphenyl moiety need to be in a sterically inaccessible coplanar conformation. Panel B: The same view with compound **1** rotated by 180° and fit into the flat density. In this orientation the morpholine moiety points toward the protein without adopting sterically unfavorable conformations within the phenoxyphenyl moiety. Panel C: The same model as in B with the 2mFo-DFc map calculated from the final model including compound **1** and showing the distinct hydrogen bonding pattern which is only possible with compound **1** bound in this orientation. The panels show amino acids that are within 5 Å of compound **1**, with the addition of N142 as it is involved in the hydrogen bonding network with the bridging water. All maps are contoured at $\sigma = 1$.
