# Supplementary material for: Crystal Structures of T. b. rhodesiense Adenosine Kinase Complexed with Inhibitor and Activator: Implications for Catalysis and Hyperactivation
Source: PLoS Negl Trop Dis. 2011 May 24;5(5):e1164. doi: 10.1371/journal.pntd.0001164 (PMC3101181; doi:10.1371/journal.pntd.0001164)
Supplement: Figure S2 — MolProbity analysis of two possible binding modes of compound 1. Panel A: When compound 1 is bound to TbrAK with the morpholine moiety pointing toward the solvent, the two phenyl rings of the phenoxyphenyl moiety need to be coplanar in order to fit the flat density. However, this conformation is sterically inaccessible as shown by large clashes in the MolProbity analysis (red and pink, Clashscore of 69.77) [1], which excludes this binding mode. Panel B: In contrast, compound 1 bound to TbrAK with the phenoxyphenyl moiety pointing towards the solvent while exhibiting a sterically favorable conformation, only minor clashes (yellow and orange, Clashscore of 23.26) between the morpholine and the pyrazole moiety are observed. This supports the conclusion that compound 1 binds to TbrAK with the phenoxyphenyl moiety pointing towards the outside and the morpholine being buried in the active site. (PDF) [file pntd.0001164.s002.pdf]

Supporting Information Figure S2

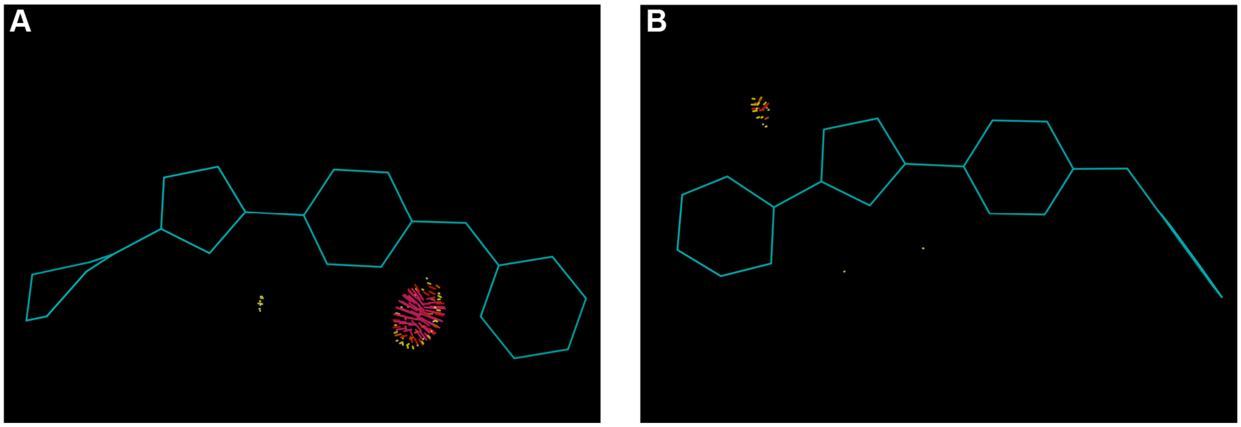

Figure S2. MolProbity analysis of two possible binding modes of compound 1. Panel A: When compound **1** is bound to TbrAK with the morpholine moiety pointing toward the solvent, the two phenyl rings of the phenoxyphenyl moiety need to be coplanar in order to fit the flat density. However, this conformation is sterically inaccessible as shown by large clashes in the MolProbity analysis (red and pink, Clashscore of 69.77) [1], which excludes this binding mode. Panel B: In contrast, compound **1** bound to TbrAK with the phenoxyphenyl moiety pointing towards the solvent while exhibiting a sterically favorable conformation, only minor clashes (yellow and orange, Clashscore of 23.26) between the morpholine and the pyrazole moiety are observed. This supports the conclusion that compound **1** binds to TbrAK with the phenoxyphenyl moiety pointing towards the outside and the morpholine being buried in the active site.

References

1. Chen VB, Arendall WB, 3rd, Headd JJ, Keedy DA, Immormino RM, et al. MolProbity: all-atom structure validation for macromolecular crystallography. Acta Crystallogr D Biol Crystallogr 66: 12-21.
