# Supplementary material for: Crystal Structures of T. b. rhodesiense Adenosine Kinase Complexed with Inhibitor and Activator: Implications for Catalysis and Hyperactivation
Source: PLoS Negl Trop Dis. 2011 May 24;5(5):e1164. doi: 10.1371/journal.pntd.0001164 (PMC3101181; doi:10.1371/journal.pntd.0001164)
Supplement: Table S1 — A comprehensive summary of all adenosine kinase structures published as of 2010, including the TbrAK structures presented in this work. (PDF) [file pntd.0001164.s003.pdf]

Table S1. A comprehensive summary of all adenosine kinase structures published as of 2010, including the TbrAK structures presented in this work.

PDB code	species	ligand	lid domain conformation
3OTX	*T. b. rhodesiense*	diadenosine pentaphosphate (AP5A)	closed
2XTB	*T. b. rhodesiense*	4-[5-(4-phenoxyphenyl)-2*H*-pyrazol-3-yl]-morpholine (compound **1**)	open
1BX4 [1]	*H. sapiens*	two adenosines	closed
2I6A [2]	*H. sapiens*	5-iodo-5'-deoxytubercidin	closed
2I6B [2]	*H. sapiens*	alkynylpyrimidine compound: 5-[4-(dimethylamino)phenyl]-6-[6-morpholin-4-ylpyridin-3-ylethynyl]pyrimidin-4-ylamine	open
1DGM [3]	*T. gondii*	adenosine	open
1LIO [4]	*T. gondii*	apo	open
1LIK [4]	*T. gondii*	two adenosines	closed
1LII [4]	*T. gondii*	adenosine, β,γ-methylene-adenosine 5'-triphosphate (AMP-PCP, a nonhydrolysable ATP analog)	closed
1LIJ [4]	*T. gondii*	7-iodotubercidin, β,γ-methylene-adenosine 5'-triphosphate (AMP-PCP, a nonhydrolysable ATP analog)	closed
2ABS [5]	*T. gondii*	β,γ-methylene-adenosine 5'-triphosphate (AMP-PCP, a nonhydrolysable ATP analog) in two different conformations still undefined density in the adenosine binding site, 6-[(4-nitrobenzyl)thio]-9-β-D-ribofuranosylpurine was added in the crystallization setup	semi-closed
2A9Y [6]	*T. gondii*	two N^6,N^6-dimethyladenosine	semi-closed
2A9Z [6]	*T. gondii*	N^6,N^6-dimethyladenosine, β,γ-methylene-adenosine 5'-triphosphate (AMP-PCP, a nonhydrolysable ATP analog)	semi-closed
2AA0 [6]	*T. gondii*	two 6-methyl mercaptopurine riboside	semi-closed
2AB8 [6]	*T. gondii*	6-methyl mercaptopurine riboside, β,γ-methylene-adenosine 5'-triphosphate (AMP-PCP, a nonhydrolysable ATP analog)	semi-closed
2PKF [7]	*M. tuberculosis*	apo	open
2PKM [7]	*M. tuberculosis*	adenosine	closed
2PKK [7]	*M. tuberculosis*	2-fluoroadenosine	closed
2PKN [7]	*M. tuberculosis*	β,γ-methylene-adenosine 5'-triphosphate (AMP-PCP, a nonhydrolysable ATP analog)	open

References

1. Mathews, II, Erion MD, Ealick SE (1998) Structure of human adenosine kinase at 1.5 Å resolution. Biochemistry 37: 15607-15620.
2. Muchmore SW, Smith RA, Stewart AO, Cowart MD, Gomtsyan A, et al. (2006) Crystal structures of human adenosine kinase inhibitor complexes reveal two distinct binding modes. J Med Chem 49: 6726-6731.
3. Cook WJ, DeLucas LJ, Chattopadhyay D (2000) Crystal structure of adenosine kinase from Toxoplasma gondii at 1.8 Å resolution. Protein Sci 9: 704-712.
4. Schumacher MA, Scott DM, Mathews, II, Ealick SE, Roos DS, et al. (2000) Crystal structures of Toxoplasma gondii adenosine kinase reveal a novel catalytic mechanism and prodrug binding. J Mol Biol 298: 875-893.
5. Zhang Y, El Kouni MH, Ealick SE (2006) Structure of Toxoplasma gondii adenosine kinase in complex with an ATP analog at 1.1 angstroms resolution. Acta Crystallogr D Biol Crystallogr 62: 140-145.
6. Zhang Y, El Kouni MH, Ealick SE (2007) Substrate analogs induce an intermediate conformational change in Toxoplasma gondii adenosine kinase. Acta Crystallogr D Biol Crystallogr 63: 126-134.
7. Reddy MC, Palaninathan SK, Shetty ND, Owen JL, Watson MD, et al. (2007) High resolution crystal structures of Mycobacterium tuberculosis adenosine kinase: insights into the mechanism and specificity of this novel prokaryotic enzyme. J Biol Chem 282: 27334-27342.
